# Heat or eat? Exploring the link between fuel poverty and diet quality among older adults aged over 50

**DOI:** 10.1093/eurpub/ckag026

**Published:** 2026-02-13

**Authors:** Huahan Yang, Adam Martin, Bryony Dawkins, Samuel Relton, Daniel Howdon

**Affiliations:** Academic Unit of Health Economics, Leeds Institute of Health Sciences, University of Leeds, Leeds, United Kingdom; Academic Unit of Health Economics, Leeds Institute of Health Sciences, University of Leeds, Leeds, United Kingdom; Academic Unit of Health Economics, Leeds Institute of Health Sciences, University of Leeds, Leeds, United Kingdom; Leeds Institute of Health Sciences, University of Leeds, Leeds, United Kingdom; Academic Unit of Health Economics, Leeds Institute of Health Sciences, University of Leeds, Leeds, United Kingdom

## Abstract

Rising fuel prices have increased the prevalence of fuel poverty, forcing households to make trade-offs between spending on energy and food, a dilemma often described as ‘heat or eat’. While prior studies have mentioned reductions in food expenditure or intake under these circumstances, less is known about the impact on overall diet quality among older adults. We analysed the association between fuel poverty and diet quality among adults aged over 50 in England using cross-sectional data from Wave 9 of the English Longitudinal Study of Ageing (ELSA; *n* = 3919). Diet quality was assessed using the Healthy Diet Indicator (HDI) score and daily fruit and vegetable intake. Fuel poverty was defined using three measures: the 10% threshold, the Low-Income High Costs (LIHC) indicator, and a subjective self-reported measure. Ordinal logistic regression was used for HDI, and ordinary least squares regression for fruit and vegetable intake. Older adults identified as fuel poor under the LIHC indicator had lower HDI scores (OR = 0.83) and consumed 0.41 fewer portions of fruit and vegetables per day compared with those not in fuel poverty. No consistent associations were observed when fuel poverty was defined using the 10% threshold or the subjective measure. Fuel poverty, when defined by the LIHC indicator, is negatively associated with diet quality in later life. Addressing fuel poverty may therefore support improved nutrition and health among older adults.

## Introduction

With the rising cost of living in the UK, more people are ‘choosing between heating and eating’, reflecting the association between fuel poverty and food [1]. Fuel poverty refers to households being unable to obtain adequate warmth at a reasonable cost, often due to a combination of low-income, high fuel prices, and poor energy efficiency in the home [2]. Living in fuel poverty has adverse effects on people’s health and well-being, including the ‘heat or eat’ dilemma, in which people struggle to heat their homes and afford adequate, nutritious food. This issue is particularly acute among older adults: according to recent estimates, ∼2.8 million older households in England are experiencing fuel poverty [3], >11 million over 50s struggled to afford energy bills [4], and >1 million people over 65 reported skipping meals to afford heating [5]. This not only limits warmth but also compromises dietary quality, as households may prioritize energy payments over purchasing sufficient or nutritious food, change cooking methods (batch cooking, slow cooking), or shift to less healthy foods (e.g. microwave-ready meals) [6]. Their vulnerability stems from both socio-economic and physiological factors: many rely on fixed pensions [7], spend more time indoors [8, 9], and face heightened health risks from cold housing [10, 11]. Crucially, this trade-off between heating and eating poses a direct threat to diet quality, which is vital for sustaining health and well-being in later life.

Given the potential impact of fuel poverty on diet and subsequent health outcomes, the method chosen to measure fuel poverty is also critical. Over time, various indicators have been developed, reflecting evolving understandings of the issue and changes in government priorities. Previously, the ‘10% indicator’ proposed by Boardman [2] was used in England, whereby a household is considered fuel poor if it needs to spend >10% of its income on fuel to maintain adequate warmth. Subsequently, this indicator was criticized for being too sensitive to fluctuations in fuel and failed to account for high-income households that could afford higher energy costs without experiencing deprivation. In 2013, the UK government introduced a new Low-Income-High-Cost (LIHC) indicator in England [12], which defined household fuel poverty as requiring energy costs above the national median, and if paying those costs would leave it with a residual income below the poverty line, an internationally recognized benchmark for relative poverty of 60% of the median national income [13]. In 2021, England adopted the Low-Income Low Energy Efficiency (LILEE) measure. Under this definition, a household is fuel poor if its income falls below the poverty line after housing and energy costs and it lives in an energy-inefficient home (EPC rating D or below). This approach emphasizes housing quality and energy efficiency more than earlier income-focused measures. In addition, some qualitative studies assess fuel poverty using subjective descriptions of cold or discomfort or objective questions about housing conditions linked to warmth or dampness. For example: ‘Being able to keep the home adequately warm (pensioners only), presence of damp (pensioners only), keeping comfortably warm in the winter, and presence of a leaking roof, damp walls/floors, damp foundations, or rotten floorboards or window frames’ [14]. ‘Do you suffer from thermal discomfort?’ or ‘Are you financially able to maintain an adequate temperature in the dwelling?’ [15] or ‘Did you feel cold during the winter?’ [16].

Alongside changes in how fuel poverty is defined, a growing body of research explores the ‘heat or eat’ dilemma, highlighting the overlapping challenges of food and energy poverty or insecurity [1]. Food poverty (or food insecurity) is commonly defined as the inability or uncertainty about the ability to obtain adequate quality or sufficient quantities of food through socially acceptable means [17]. Early research on ‘heating or eating’ was predominantly conducted in the United States. Bhattacharya *et al.* [18] found that poor families reduced their food expenditure in winter to cope with the cold weather, and the calorie intake of adults and children decreased by 10% in winter, whereas rich families did not reduce it. Frank *et al.* [19] found that households participating in a Low-Income Home Energy Assistance Program reported more severe food insecurity overall; however, children in households not receiving assistance faced greater risks of nutrition problems for growth compared to those in participating households. Nord and Kentor [20] found that food insecurity among low-income elderly households in the US varied significantly by season, particularly in regions with high winter heating and summer cooling costs. In States with high heating expenses, food insecurity rates were notably higher in the winter compared to the summer. These findings indicate that fluctuations in energy demand during the winter can intensify financial vulnerability and lead to poorer food quality among low-income households.

Some evidence has also been found in UK-based studies. In a qualitative study based on semi-structured interviews with ten older women in the UK, several participants reported that maintaining adequate heating was more important than food [7]. Beatty *et al.* [21] found that the most poor elderly households had difficulty paying for fuel in winter and that food expenses were significantly reduced during colder winters. Snell *et al.* [14] found that in fuel-poor households, neither maintaining warmth nor obtaining adequate food can be reliably maintained, indicating that food and energy expenditures are also affected by other factors and are not simply substitutes. Burlinson *et al.* [22] found that people who used prepayment metres had lower vegetable and fruit consumption than those who used post-payment energy bill payment methods.

Although research on the ‘heat or eat’ dilemma has grown in recent years, some notable limitations remain. First, much of the existing literature has focused narrowly on food expenditure or the consumption of specific food groups, such as fruits and vegetables. In this study, we employ the Healthy Diet Indicator (HDI) as the outcome measure, a widely used and comprehensive tool for assessing diet quality. Second, unlike many previous studies, we explicitly examine fuel poverty as a distinct financial indicator using established definitions (the 10% threshold and the LIHC indicator), alongside a subjective measure of fuel poverty. This approach enables a more accurate and targeted assessment of the relationship between fuel poverty and diet quality. Third, while most studies of the ‘heat or eat’ trade-off focus on the general population, our study specifically targets older adults, a group that may be particularly vulnerable to fuel poverty due to fixed incomes and higher energy needs.

Using nationally representative data from the English Longitudinal Study of Ageing (ELSA), this study investigates the association between fuel poverty and diet quality among older adults in England, considering both overall diet quality (Healthy Diet Indicator, HDI) and total fruit and vegetable intake.

## Methods

### Data

The data for this study were obtained from Wave 9 (July 2018–July 2019) of the English Longitudinal Study of Ageing (ELSA), a nationally representative sample of adults aged 50 and over in England. We selected this wave because it was the first to provide comprehensive dietary information for participants, including a 24-hour dietary recall survey completed by 5068 of the 8736 total participants.

Wave 9 also includes interviewer-administered survey data on household income, expenditure on food, electricity, gas, and other fuels, as well as participants’ demographic and socioeconomic characteristics (age, gender, marital status, ethnicity, education, and work status) and information on accommodation problems (e.g. being too cold in winter, water leaks, rising damp, and electrical or plumbing issues). After removing outliers and cases with missing values, the final sample for the main analysis comprised 3919 respondents.

We developed an empirical model using three definitions of fuel poverty to explore its association with diet quality. To account for heterogeneity among adults aged 50 and above, we also conducted subgroup analyses by age groups (50–59, 60–69, 70–79, and 80–90).

### Variables

Two different indicators of diet quality are used in this study and employed as outcomes.

#### Healthy diet indicator (HDI)

The Healthy Diet Indicator (HDI) was developed to assess overall diet quality based on the World Health Organization’s (WHO) dietary recommendations for the prevention of chronic diseases [16]. The HDI sums scores across individual food groups and nutrients, awarding one point for each criterion in which a participant’s intake falls within the recommended range. In this study, we used the version of the HDI developed by Kanauchi and Kanauchi [23], which includes minor modifications to the original WHO indicator. For nutrients without clear WHO thresholds (dietary fibre, unsaturated fat, and potassium), cut-offs were taken from FAO reports and earlier WHO guidelines. Trans fatty acids and salt intake were excluded due to data limitations.

The HDI applied here included seven components: ≥400 g/day of fruit and vegetables, <30% of total energy from fat, <10% of total energy from saturated fatty acids, 6%–11% of total energy from polyunsaturated fatty acids, <10% of total energy from free sugar, ≥25 g/day dietary fibre, and ≥3500 mg/day potassium. Participants received one point for each criterion met, yielding a total score ranging from 0 to 7. This HDI version has been used in previous UK studies [24], supporting its suitability for assessing diet quality in the current sample.

#### Total fruit and vegetable consumption

The indicator of assessing fruit and vegetable intake is a continuous variable and is derived from the nutrition section of the ELSA wave 9 and is described as: ‘Fruit & Veg intake portion/day’.

### Indicator of fuel poverty

Three different measures are used in this study. Objective measures are usually based on the relationship between income and fuel expenditure. This study used 10% definition and LIHC as indicators for assessing fuel poverty and regards them as binary variables according to their assessment criteria:

1. The 10% definition indicator was coded as 1 if the participant’s household spends >10% of household income on fuel and 0 otherwise.
2. The LIHC indicator was assigned a value of 1 in households where (i) required fuel costs are above the national median level and (ii) the residual household net income is below the official poverty line (i.e. 60% of the national median income) after deducting energy expenses. Otherwise, it was coded as 0. To ensure a fair comparison between different households, household income was equivalized by using the OECD equivalence scale, and fuel expenditure was equivalized according to fuel cost equivalization standards presented in Hills [13].
3. Subjective assessments were conducted by asking households questions about their ability to afford fuel or keep their homes warm, as well as their housing conditions. In this study, we selected a question from wave 9 of ELSA data survey: ‘Does your accommodation have any of these problems?’ (potential answers detailed in Table S1). Selecting the answer that it was ‘too cold in winter’ was used as an indicator of fuel poverty. This subjective indicator was coded as 1 when the participants selected this answer and coded as 0 otherwise.

### Covariates

In our analysis, we controlled for a range of demographic and socioeconomic characteristics that are also identified in other literature and that are collected in wave 9 of ELSA [14, 21, 22]. These are: gender, age, ethnicity, marital status, educational attainment, and work status. We also controlled for annual food expenditure and problems in accommodation (all variable definitions can be found in Supplementary Table S1).

### Analysis

#### Association between fuel poverty and diet quality (HDI)

Diet quality was measured using the Healthy Diet Indicator (HDI) score, which ranges from 0 to 7. As higher scores indicate better overall diet quality, we coded it as an ordinal variable and applied ordinal logistic regression to assess its association with fuel poverty. Analyses were conducted separately for the three fuel poverty indicators and were estimated in three stages: Model 1 (unadjusted), Model 2 (adjusted for demographic and socioeconomic characteristics), and Model 3 (additionally adjusted for housing problems and annual food expenditure).

#### Association between fuel poverty and total fruit and vegetable intake

As total fruit and vegetable intake is recorded as daily portion intake, we treated it as a continuous variable and analysed its relationship with fuel poverty using ordinary least squares (OLS) regression. For this outcome, we estimated fully adjusted models including demographic, socioeconomic, housing, and food expenditure covariates. Separate regressions were run for each of the three fuel poverty indicators.

## Results

Table 1 presents the demographic and socioeconomic characteristics of the study sample, as well as the distribution and prevalence of fuel poverty according to three measurement approaches. The analytical sample consisted of 3919 participants aged 50–90 years, of whom 45.6% were male and 54.4% were female. The prevalence of fuel poverty varied by measurement approach: 13.9% of participants were classified as fuel poor using the 10% threshold, 10.8% using the LIHCs indicator, and 3.7% reported feeling cold at home according to the subjective measure. Similar discrepancies between subjective and objective measures have been documented in previous studies [25], suggesting that the distributions observed in our sample are consistent with prior research.

**Table 1.** Demographic, socioeconomic, and fuel poverty characteristics of the study sample

Variable	*N* (%)/mean (SD)/range
Sample size	3919
Fuel poverty (10% threshold)	–
No	3414 (87.1)
Yes	505 (13.9)
Fuel poverty (LIHC)	–
No	3496 (89.2)
Yes	423 (10.8)
Feel cold (subjective indicator)	–
No	3774 (96.3)
Yes	145 (3.7)
Variables	
Age	68.5 (SD: 7.4); range: 50–90
Gender	–
Male	1786 (45.6)
Female	2133 (54.4)
Ethnicity	–
White	3820 (97.5)
Non-White	99 (2.5)
Education	–
Nvq4/nvq5/degree or equivalent	1191 (30.4)
Higher education below degree	708 (18.1)
Nvq3/gce a level equivalent	440 (11.2)
Nvq2/gce o level equivalent	829 (21.2)
Nvq1/cse other grade equivalent	113 (2.9)
Uncategorized qualification	256 (6.5)
No qualification	382 (9.7)
Marital status	–
Single	212 (5.4)
Married	2463 (62.8)
Remarried	465 (11.9)
Separated	38 (1.0)
Divorced	397 (10.1)
Widowed	344 (8.8)
Whether in paid employment	–
No	2798 (71.4)
Yes	1121 (28.6)
Yearly spend on food and groceries (GBP, thousands)	4.32 (SD: 2.50)
Problems in accommodation (include cold, water leaks, rising damp, electrical or plumbing problems)	–
No	3592 (91.7)
Yes	327 (8.3)
Fruits and vegetables intake (last 24 hours: portion)	4.73 (SD: 3.89)

The distribution of participants across Healthy Diet Indicator (HDI) score categories is shown in Table S2. The majority of participants were concentrated in the lower HDI score categories, with progressively fewer participants in the higher score categories.

### The results of fuel poverty and the healthy diet indicator (HDI) score

Table 2 presents the results of ordinal logistic regression models examining the association between fuel poverty, measured using the 10% threshold, and diet quality (HDI score). In the unadjusted model (Model 1), individuals experiencing fuel poverty had 17% lower odds of being in a higher HDI category compared with those not in fuel poverty (OR = 0.83). After adjustment for demographic and socioeconomic characteristics (sex, age, ethnicity, education, employment, and marital status) in Model 2, the association remained negative (OR = 0.86) but was no longer statistically significant. Further adjustment for housing problems and food expenditure in Model 3 yielded similar effect estimates (OR = 0.86), with the association remaining statistically non-significant.

**Table 2.** Ordinal logistic regression results of fuel poverty (10% indicator) and HDI score

	Model 1		Model 2		Model 3	
	OR	95% CI	OR	95% CI	OR	95% CI
Fuel poverty	–	–	–	–	–	–
No	Ref.	–	Ref.	–	Ref.	–
Yes	0.83	(0.71–0.98)	0.86	(0.74–1.02)	0.86	(0.73–1.02)
Age	–	–	1.00	(0.99–1.00)	1.00	(0.99–1.00)
Gender	–	–	–	–	–	–
Male	–	–	Ref.	–	Ref.	–
Female	–	–	0.96	(0.86–1.08)	0.97	(0.59–1.20)
Ethnicity	–	–	–	–	–	–
Non-White	–	–	Ref.	–	Ref.	–
White	–	–	0.84	(0.59–1.20)	0.84	(0.59–1.20)
Education	–	–	–	–	–	–
Nvq4/nvq5/degree or equivalent	–	–	Ref.	–	Ref.	–
Higher education below degree	–	–	0.90	(0.76–1.06)	0.91	(0.77–1.07)
Nvq3/gce a level equivalent	–	–	0.94	(0.77–1.15)	0.95	(0.78–1.15)
Nvq2/gce o level equivalent	–	–	0.89	(0.76–1.04)	0.90	(0.76–1.05)
Nvq1/cse other grade equivalent	–	–	0.97	(0.69–1.35)	0.98	(0.70–1.38)
Uncategorized qualification	–	–	0.81	(0.63–1.03)	0.82	(0.64–1.04)
No qualification	–	–	0.62	(0.50–0.76)	0.63	(0.51–0.78)
Marital status	–	–	–	–	–	–
Single	–	–	Ref.	–	Ref.	–
Married	–	–	1.36	(1.05–1.74)	1.31	(1.01–1.68)
Remarried	–	–	1.31	(0.98–1.75)	1.26	(0.95–1.69)
Separated	–	–	1.19	(0.63–2.25)	1.20	(0.64–2.26)
Divorced	–	–	1.16	(0.86–1.56)	1.16	(0.86–1.57)
Widowed	–	–	1.41	(1.03–1.94)	1.41	(1.03–1.93)
Whether in paid employment	–	–	–	–	–	–
No	–	–	Ref.	–	Ref.	–
Yes	–	–	1.01	–	1.00	(0.87–1.16)
Food expenditure	–	–	–	–	1.02	(1.00–1.05)
Problems in accommodation	–	–	–	–	–	–
No	–	–	–	–	Ref.	–
Yes	–	–	–	–	0.90	(0.73–1.10)

Table 3 presents the results of ordinal logistic regression models examining the association between fuel poverty, measured using the LIHC indicator, and diet quality. In the unadjusted model (Model 1), individuals experiencing fuel poverty had 18% lower odds of being in a higher HDI category compared with those not in fuel poverty (OR = 0.82). This association was attenuated and no longer statistically significant after adjustment for demographic and socioeconomic characteristics (Model 2). However, after further adjustment for housing problems and food expenditure (Model 3), the association re-emerged, with individuals in fuel poverty having 17% lower odds of achieving a higher HDI score compared with those not in fuel poverty.

**Table 3.** Ordinal logistic regression results of fuel poverty (LIHC) and HDI score

	Model 1		Model 2		Model 3	
	OR	95% CI	OR	95% CI	OR	95% CI
Fuel poverty	–	–	–	–	–	–
No	Ref.	–	Ref.	–	Ref.	–
Yes	0.82	(0.69–0.98)	0.84	(0.71–1.01)	0.83	(0.70–1.00)
Age	–	–	1.00	(0.99–1.01)	1.00	(0.99–1.01)
Gender	–	–	–	–	–	–
Male	–	–	Ref.	–	Ref.	–
Female	–	–	0.96	(0.86–1.08)	0.97	(0.86–1.08)
Ethnicity	–	–	–	–	–	–
Non-White	–	–	Ref.	–	Ref.	–
White	–	–	0.84	(0.59–1.20)	0.84	(0.59–1.20)
Education	–	–	–	–	–	–
Nvq4/nvq5/degree or equivalent	–	–	Ref.	–	Ref.	–
Higher education below degree	–	–	0.90	(0.77–1.07)	0.91	(0.77–1.07)
Nvq3/gce a level equivalent	–	–	0.95	(0.78–1.15)	0.95	(0.78–1.16)
Nvq2/gce o level equivalent	–	–	0.89	(0.76–1.04)	0.90	(0.77–1.06)
Nvq1/cse other grade equivalent	–	–	0.98	(0.70–1.37)	1.00	(0.71–1.41)
Uncategorized qualification	–	–	0.82	(0.64–1.04)	0.82	(0.65–1.05)
No qualification	–	–	0.62	(0.50–0.76)	0.63	(0.51–0.78)
Marital status	–	–	–	–	–	–
Single	–	–	Ref.	–	Ref.	–
Married	–	–	1.37	(1.06–1.76)	1.31	(1.02–1.69)
Remarried	–	–	1.32	(0.99–1.76)	1.27	(0.95–1.70)
Separated	–	–	1.19	(0.63–2.25)	1.20	(0.64–2.26)
Divorced	–	–	1.16	(0.86–1.56)	1.16	(0.86–1.57)
Widowed	–	–	1.41	(1.03–1.93)	1.41	(1.03–1.92)
Whether in paid employment	–	–	–	–	–	–
No	–	–	Ref.	–	Ref.	–
Yes	–	–	1.01	(0.88–1.17)	1.01	(0.87–1.16)
Food expenditure	–	–	–	–	1.02	(1.00–1.05)
Problems in accommodation	–	–	–	–	–	–
No	–	–	–	–	Ref.	–
Yes	–	–	–	–	0.89	(0.73–1.10)

Table 4 shows the results of the ordinal logistic regression models examining the association between the subjective measure of fuel poverty (‘feeling cold at home’) and HDI scores. Across all models, there was no statistically significant relationship between subjective fuel poverty and diet quality. In the unadjusted model (Model 1), the odds ratio was 0.95, and in the fully adjusted model (Model 3) it shifted to 1.16, but in both cases the estimates were small and highly uncertain. Overall, these results provide little evidence that perceived cold at home is associated with diet quality.

**Table 4.** Ordinal logistic regression results of subjective measurement of fuel poverty and HDI score

	Model 1		Model 2		Model 3	
	OR	95% CI	OR	95% CI	OR	95% CI
Fuel poverty	–	–	–	–	–	–
No	Ref.	–	Ref.	–	Ref.	–
Yes	0.95	(0.71–1.28)	0.97	(0.72–1.31)	1.16	(0.78–1.72)
Age	–	–	1.00	(0.99–1.01)	1.00	(0.99–1.01)
Gender	–	–	–	–	–	–
Male	–	–	Ref.	–	Ref.	–
Female	–	–	0.96	(0.86–1.08)	0.97	(0.86–1.09)
Ethnicity	–	–	–	–	–	–
Non-white	–	–	Ref.	–	Ref.	–
White	–	–	0.85	(0.59–1.21)	0.85	(0.59–1.22)
Education	–	–	–	–	–	–
Nvq4/nvq5/degree or equivalent	–	–	Ref.	–	Ref.	–
Higher education below degree	–	–	0.90	(0.76–1.06)	0.90	(0.76–1.06)
Nvq3/gce a level equivalent	–	–	0.94	(0.77–1,14)	0.94	(0.77–1.15)
Nvq2/gce o level equivalent	–	–	0.88	(0.75–1.03)	0.89	(0.76–1.04)
Nvq1/cse other grade equivalent	–	–	0.95	(0.68–1.34)	0.97	(0.69–1.36)
Uncategorized qualification	–	–	0.81	(0.63–1.03)	0.81	(0.64–1.04)
No qualification	–	–	0.61	(0.49–0.75)	0.62	(0.50–0.76)
Marital status	–	–	–	–	–	–
Single	–	–	Ref.	–	Ref.	–
Married	–	–	1.36	(1.06–1.75)	1.32	(1.02–1.70)
Remarried	–	–	1.31	(0.98–1.75)	1.27	(0.95–1.71)
Separated	–	–	1.20	(0.64–2.26)	1.22	(0.64–2.29)
Divorced	–	–	1.15	(0.86–1.56)	1.16	(0.86–1.57)
Widowed	–	–	1.42	(1.03–1.94)	1.41	(1.03–1.93)
Whether in paid employment	–	–	–	–	–	–
No	–	–	–	–	Ref.	–
Yes	–	–	–	–	1.02	0.88–1.17)
Food expenditure	–	–	–	–	–	(1.00–1.05)
Problems in accommodation	–	–	–	–	–	–
No	–	–	–	–	Ref.	–
Yes	–	–	–	–	0.83	(0.64–1.09)

### The results of fuel poverty and total fruit and vegetable intake

Supplementary Table S3 presents the results of OLS regressions examining the association between fuel poverty and total daily fruit and vegetable intake across the three indicators of fuel poverty. When fuel poverty was defined using the 10% threshold, participants experiencing fuel poverty consumed on average 0.27 fewer daily portions of fruit and vegetables compared with those not experiencing fuel poverty, although the difference was not statistically significant. Using the LIHC indicator, participants in fuel poverty consumed on average 0.41 fewer daily portions than those not in fuel poverty. In contrast, under the subjective measure of fuel poverty, the association was positive (+0.53 daily portions) but not statistically significant.

Results of the subgroup analyses by age group are presented in Tables S4 and S5.

## Discussion

In this study, we examined the association between fuel poverty and diet quality among older adults aged over 50 using data from ELSA wave 9. When fuel poverty was measured by the LIHCs indicator, we found that individuals experiencing fuel poverty had 17% lower odds of achieving higher HDI scores and consumed 0.41 fewer daily portions of fruit and vegetables than those not in fuel poverty. In the unadjusted model, fuel poverty was significantly associated with lower diet quality. The association was weakened after adjusting for demographic and socioeconomic characteristics, suggesting that these factors explain part of the relationship. This pattern should be interpreted cautiously, as the cross-sectional design cannot fully separate the independent effects of fuel poverty from demographic and socioeconomic factors, nor establish the direction of these associations. However, the association re-emerged after further adjustment for housing problems and food expenditure, suggesting that these factors help to clarify the association between fuel poverty and diet quality.

Compared to previous studies, this study found a similar trend: in the ‘heat or eat’ trade-off, households experiencing financial hardship are negatively impacted by their eating or food intake. Our study extends this literature by using a comprehensive measure of diet quality and explicitly assessing fuel poverty to examine its relationship with overall diet quality. By applying the HDI, we were able to capture overall dietary quality rather than focusing on single components of diet, providing a broader assessment of the nutritional consequences of fuel poverty. Moreover, instead of limiting the analysis to low-income groups, we applied three different indicators of fuel poverty, capturing its multidimensional nature. Among the results, the LIHC indicator provided clearer evidence of the link between fuel poverty and diet quality, extending the understanding of the ‘heat or eat’ dilemma beyond expenditure patterns to overall nutritional outcomes.

This study also has notable strengths. To our knowledge, this is the first attempt to assess the link between overall diet quality and fuel poverty. This study uses the ninth wave of ELSA data which provides a robust and representative data sample for this analysis: it included demographic and socioeconomic characteristics, along with detailed information on the participants’ fuel expenses. In addition, the ninth wave of ELSA provided detailed dietary information, which is sufficient to assess overall diet quality as well as the consumption of specific food groups.

Several limitations should be noted. The study used a single wave of ELSA, so causal relationships cannot be established. The sample included adults aged over 50 living in England, most of whom lived alone, as a couple, or with non-dependent children; very few lived with dependent children (∼1%). Therefore, the sample in this study differs significantly from the typical care responsibilities of families with young children. A further consideration relates to dietary assessment. The ninth wave of ELSA collected dietary data using the Oxford WebQ, a validated 24-hour recall tool widely used in large-scale studies. This instrument provides a comprehensive and standardized measure of diet, which is a key strength of the dataset. However, it captures short-term intake and may not fully reflect long-term dietary patterns. In terms of the subgroup analyses, by age group (supplementary material), although the point estimates suggest that the association between fuel poverty and diet quality may strengthen with age, the smaller sample sizes within each subgroup led to wider confidence intervals and reduced statistical power, limiting our ability to draw firm or meaningful conclusions from these analyses. These patterns should be interpreted cautiously but may indicate age-related differences that warrant investigation in future studies using larger samples or longitudinal data. Finally, our data were collected prior to the COVID-19 pandemic and the cost-of-living crisis, so the findings reflect patterns of fuel poverty and diet quality during a baseline period. This provides a useful reference point for understanding changes over time and allows future research to examine how the pandemic and subsequent increases in energy prices may have affected older adults’ experiences of fuel poverty and dietary outcomes.

These findings suggest that policies aimed at reducing fuel poverty, such as improving household energy efficiency, stabilizing energy costs, or providing targeted financial support, may also yield important co-benefits for dietary quality and nutritional health among older adults.

## Supplementary Material

ckag026_Supplementary_Data

## Data Availability

The data analysed in this study are from the English Longitudinal Study of Ageing (ELSA). These data are publicly available via the UK Data Service to registered users, subject to the relevant data access agreements. Key Points Rising fuel prices may force households into a ‘heat or eat’ dilemma, with implications for nutrition in older adults. This study examined fuel poverty and diet quality using wave 9 of the English Longitudinal Study of Ageing. Diet quality was measured using both the Healthy Diet Indicator (HDI) and daily fruit and vegetable intake. Fuel poverty defined by the Low-Income High Costs (LIHC) indicator was significantly associated with poorer diet quality. Policies addressing fuel poverty could contribute to improving nutrition and health among older adults.
