# Supplementary material for: Heat or eat? Exploring the link between fuel poverty and diet quality among older adults aged over 50
Source: Eur J Public Health. 2026 Feb 13;36(3):ckag026. doi: 10.1093/eurpub/ckag026 (PMC13230498; doi:10.1093/eurpub/ckag026)
Supplement: ckag026_Supplementary_Data [file ckag026_supplementary_data.docx]

**Supplementary Material**

Table S1: Definition of dependent variables

| **Definition of dependent variables** | |
| --- | --- |
| **Variables** | **Description** |
| HDI score | An ordinal variable of the Healthy Diet Indicator evaluation score from 0 to 7 points |
| Intake of fruit and vegetables | Total intake of fruit and vegetables within the last 24 hours (unit: portion) |
| **Definition of fuel poverty variables** | |
| **Variables** | **Description** |
| Fuel poverty-10% definition | If a household spends more than 10% of its household income on fuel  =0 if the household spend ≤ 10% of income  =1 if the household spend > 10% of income |
| Fuel Poverty-LIHC (Low Income High Cost) definition | 1) Household required fuel costs are above the national median level; and 2) After deducting energy expenses, the residual household net income is below the official poverty line  =1 if the above conditions are met, =0 otherwise |
| Fuel Poverty- Subjective Method | Question: “Does your accommodation have any of these (too cold in winter/ water leaks from roof/gutters/windows/rising damp and electrical or plumbing problems) problems?”  Participants answered “Too cold in winter” of their accommodation.  =0 if the participants did not tick “Too cold in winter”.  =1 if the participants tick “Too cold in winter” |
| **Definition of covariates** | |
| **Variables** | **Description** |
| Gender | A binary variable, 0=Male, 1=Female |
| Age | A continuous variable. Age of participants. |
| Ethnicity | A binary variable, 0=Non-white, 1=White |
| Education | A categorical variable includes: nvq1/CSE other grade equivalent; nvq2/GCE O level equivalent; nvq3/GCE A level equivalent; nvq4/nvq5/degree or equivalent; Higher education below degree; Foreign/other. |
| Marriage | A categorical variable includes: single, married, remarried, separated, divorced, and widowed. |
| Work status | A binary variable. 0=Not in paid employment, 1=in paid employment. |
| Food expenditure | A continuous variable. Annual expenditure on food per household (unit: thousands of pounds) |
| House problems | A binary variable.  Rising damp, water leaks from roof/gutters/windows, and electrical or plumbing problems of participants’ accommodation were included.  =1 if participants tick any of the accommodation problems above  =0 if participants did not tick any of the accommodation problems above. |

Table S2: Distribution of participants by Healthy Diet Indicator (HDI) score

| **HDI score** | **Male (%)** | **Female (%)** | **N (%)** |
| --- | --- | --- | --- |
| 0 | 278 (15.6) | 343 (16.1) | 621 (15.8) |
| 1 | 449 (25.1) | 572 (26.8) | 1021 (26.1) |
| 2 | 484 (27.1) | 557 (26.1) | 1041 (26.6) |
| 3 | 330 (18.5) | 393 (18.4) | 723 (18.4) |
| 4 | 192 (10.8) | 195 (9.1) | 387 (9.9) |
| 5 | 45 (2.5) | 62 (2.9) | 107 (2.7) |
| 6 | 8 (0.4) | 11 (0.5) | 19 (0.5) |
| 7 | 0 | 0 | 0 |
| **N** | 1786 | 2133 | 3919 |

Table S3: OLS regression results of fruits and vegetables intake and three different measures of fuel poverty

|  | **10% threshold** | | **LIHC** | | **Subjective measurement** | |
| --- | --- | --- | --- | --- | --- | --- |
|  | **Coefficient** | **95% CI** | **Coefficient** | **95% CI** | **Coefficient** | **95% CI** |
| **Intercept** | 3.15 | (1.51, 4.79) | 3.16 | (1.52, 4.80) | 3.00 | (1.37, 4.64) |
| **Fuel poverty** |  |  |  |  |  |  |
| No | Ref. |  | Ref. |  | Ref. |  |
| Yes | -0.27 | (-0.64, 0.09) | -0.41 | (-0.81, -0.02) | 0.53 | (-0.32, 1.37) |
| **Age** | 0.02 | (0.00, 0.04) | 0.02 | (0.00, 0.04) | 0.02 | (0.00, 0.42) |
| **Gender** |  |  |  |  |  |  |
| Male | Ref. |  | Ref. |  | Ref. |  |
| Female | 0.96 | (0.71, 1.21) | 0.95 | (0.70, 1.20) | 0.95 | (0.70, 1.21) |
| **Ethnicity** |  |  |  |  |  |  |
| Non-White | Ref. |  | Ref. |  | Ref. |  |
| White | -0.30 | (-1.07, 0.48) | -0.29 | (-1.07, 0.48) | -0.26 | (-1.03, 0.52) |
| **Education** |  |  |  |  |  |  |
| Nvq4/nvq5/degree or equivalent | Ref. |  | Ref. |  | Ref. |  |
| Higher ed below degree | -0.28 | (-0.64, 0.08) | -0.27 | (-0.63, 0.09) | -0.29 | (-0.65, 0.07) |
| Nvq3/gce a level equivalent | -0.37 | (-0.79, 0.06) | -0.35 | (-0.78, 0.07) | -0.38 | (-0.80, 0.04) |
| Nvq2/gce o level equivalent | -0.48 | (-0.83, -0.14) | -0.47 | (-0.81, -0.12) | -0.50 | (-0.85, -0.16) |
| Nvq1/CSE other grade equivalent | -0.62 | (-1.37, 0.13) | -0.58 | (-1.33, 0.17) | -0.66 | (-1.40, 0.09) |
| Foreign/other | -0.74 | (-1.27, -0.22) | -0.72 | (-1.24, -0.19) | -0.75 | (-1.28, -0.23) |
| No qualification | -1.45 | (-1.90, -1.00) | -1.43 | (-1.89, -0.98) | -1.48 | (-1.93, -1.03) |
| **Marital status** |  |  |  |  |  |  |
| Single | Ref. |  | Ref. |  | Ref. |  |
| Married | 0.17 | (-0.38, 0.71) | 0.18 | (-0.37, 0.72) | 0.19 | (-0.36, 0.73) |
| Remarried | 0.04 | (-0.59, 0.67) | 0.05 | (-0.58, 0.68) | 0.06 | (-0.58, 0.69) |
| Separated | -0.47 | (-1.80, 0.86) | -0.48 | (-1.81, 0.85) | -0.46 | (-1.79, 0.88) |
| Divorced | 0.13 | (-0.51, 0.78) | 0.13 | (-0.51, 0.78) | 0.13 | (-0.51, 0.78) |
| Widowed | 0.37 | (-0.31, 1.05) | 0.37 | (-0.31, 1.04) | 0.38 | (-0.30, 1.06) |
| **Whether in paid employment** |  |  |  |  |  |  |
| No | Ref. |  | Ref. |  | Ref. |  |
| Yes | -0.11 | (-0.42, 0.20) | -0.10 | (-0.41, 0.21) | -0.10 | (-0.41, 0.21) |
| **Food expenditure** | 0.05 | (0.00, 0.10) | 0.05 | (0.00, 0.10) | 0.04 | (0.00, 0.09) |
| **Problems in accommodation** |  |  |  |  |  |  |
| No | Ref. |  | Ref. |  | Ref. |  |
| Yes | -0.20 | (-0.64, 0.24) | -0.20 | (-0.64, 0.24) | -0.45 | (-1.02, 0.13) |

Table S4: Association between fuel poverty and diet quality stratified by age group

|  | **10% threshold** | | **LIHC** | | **Subjective measurement** | |
| --- | --- | --- | --- | --- | --- | --- |
|  | **OR** | **95% CI** | **OR** | **95% CI** | **OR** | **95% CI** |
| **Fuel poverty** | | | | | | |
| Age: 50-59 | | | | | | |
| No | Ref. |  | Ref. |  | Ref. |  |
| Yes | 0.83 | (0.49, 1.39) | 0.86 | (0.53, 1.40) | 1.98 | (0.77, 5.10) |
| Age: 60-69 | | | | | | |
| No | Ref. |  | Ref. |  | Ref. |  |
| Yes | 0.79 | (0.63, 1.01) | 0.75 | (0.58, 0.97) | 1.04 | (0.59, 1.83) |
| Age: 70-79 | | | | | | |
| No | Ref. |  | Ref. |  | Ref. |  |
| Yes | 0.96 | (0.70, 1.31) | 0.93 | (0.65, 1.34) | 0.83 | (0.38, 1.80) |
| Age: 80+ | | | | | | |
| No | Ref. |  | Ref. |  | Ref. |  |
| Yes | 1.34 | (0.76, 2.35) | 1.59 | (0.84, 3.01) | 4.86 | (0.65, 36.02) |

Table S5: Association between fuel poverty and intake of fruit and vegetables stratified by age group

|  | **10% threshold** | | **LIHC** | | **Subjective measurement** | |
| --- | --- | --- | --- | --- | --- | --- |
|  | **Coefficient** | **95% CI** | **Coefficient** | **95% CI** | **Coefficient** | **95% CI** |
| **Fuel poverty** | | | | | | |
| Age: 50-59 | | | | | | |
| No | Ref. |  | Ref. |  | Ref. |  |
| Yes | -0.68 | (-1.62, 0.26) | -0.63 | (-1.53, 0.28) | -0.10 | (-1.89, 1.68) |
| Age: 60-69 | | | | | | |
| No | Ref. |  | Ref. |  | Ref. |  |
| Yes | -0.29 | (-0.84, 0.26) | -0.46 | (-1.05, 0.14) | 0.47 | (-0.85, 1.78) |
| Age: 70-79 | | | | | | |
| No | Ref. |  | Ref. |  | Ref. |  |
| Yes | -0.14 | (-0.80, 0.53) | -0.26 | (-1.02, 0.51) | 0.71 | (-0.84, 2.26) |
| Age: 80+ | | | | | | |
| No | Ref. |  | Ref. |  | Ref. |  |
| Yes | 0.68 | (-0.43, 1.79) | 0.68 | (-0.59, 1.94) | 2.32 | (-1.71, 6.35) |
